# Soy-Based Infant Formula Feeding and Ultrasound-Detected Uterine Fibroids among Young African-American Women with No Prior Clinical Diagnosis of Fibroids

**DOI:** 10.1289/ehp.1510082

**Published:** 2015-11-13

**Authors:** Kristen Upson, Quaker E. Harmon, Donna D. Baird

**Affiliations:** Epidemiology Branch, National Institute of Environmental Health Sciences, National Institutes of Health, Department of Health and Human Services, Research Triangle Park, North Carolina, USA

## Abstract

**Background:**

Early-life soy phytoestrogen exposure has been shown in Eker rats to increase uterine fibroid incidence in adulthood. Two large epidemiologic cohorts have provided some support for increased fibroid risk with infant soy formula feeding in women, but both cohorts relied on self-report of clinically diagnosed fibroids.

**Objective:**

We evaluated the relationship between infant soy formula feeding and ultrasound-detected fibroids.

**Methods:**

The Study of Environment, Lifestyle & Fibroids (SELF) is an ongoing cohort study of 1,696 African-American women ages 23–34 years with baseline ultrasound screening to detect and measure fibroids ≥ 0.5 cm in diameter. Questionnaire data on soy formula feeding during infancy was ascertained for 1,553 participants (89% based on mother’s report), of whom 345 were found to have fibroids. We estimated the association between soy formula feeding and fibroid prevalence and tumor number using log-binomial regression. Among those with fibroids, we compared fibroid size between soy formula-exposed and unexposed women using multivariable linear regression.

**Results:**

We did not observe an association between soy formula feeding and fibroid prevalence [adjusted prevalence ratio (aPR) 0.9, 95% CI: 0.7, 1.3]. Nor were exposed women with fibroids more likely to have ≥ 2 tumors than unexposed women with fibroids (aPR 1.0, 95% CI: 0.7, 1.6). However, exposed women with fibroids had significantly larger fibroids than unexposed women with fibroids. On average, soy formula feeding was associated with a 32% increase in the diameter of the largest fibroid (95% CI: 6%, 65%) and a 127% increase in total tumor volume (95% CI: 12%, 358%).

**Conclusions:**

Our observation that women fed soy formula as infants have larger fibroids than unexposed women provides further support for persistent effects of early life phytoestrogen exposure on the uterus.

**Citation:**

Upson K, Harmon QE, Baird DD. 2016. Soy-based infant formula feeding and ultrasound-detected uterine fibroids among young African-American women with no prior clinical diagnosis of fibroids. Environ Health Perspect 124:769–775; http://dx.doi.org/10.1289/ehp.1510082

## Introduction

Uterine fibroids, or leiomyomata, are hormone-dependent benign tumors of the uterine smooth muscle that appear after menarche and generally regress after menopause (Ross et al. 1986; Samadi et al. 1996; Templeman et al. 2009). Uterine fibroids are associated with substantial morbidity, including heavy menstrual bleeding and pelvic pain, and are the leading indication for hysterectomy in the United States (Merrill 2008; Stewart 2001). This condition develops in most reproductive-age women, with a reported cumulative incidence exceeding 80% for African-American women and reaching 70% for white women by age 50 years (Baird et al. 2003). Despite the adverse personal and public health consequences of this common condition, the etiology of uterine fibroids remains unclear.

Infancy may be a critical window for uterine fibroid development because uterine smooth muscle development begins *in utero* and continues postnatally until puberty. It has been postulated that early hormonal exposure during periods of development may result in developmental reprogramming via epigenetic changes that persist in adulthood, leading to disease onset or progression (Jirtle and Skinner 2007; Walker 2011). One such exogenous hormonal exposure that may lead to uterine fibroid development is soy-based infant formula feeding. Infant soy formula contains phytoestrogens, predominantly the isoflavones genistein and daidzein, that are structurally similar to estradiol and can interact with estrogen receptors, although weakly compared to estradiol (Jefferson et al. 2012; McCarver et al. 2011; Woods 2003). A laboratory study of Eker rats demonstrated that postnatal genistein exposure increased fibroid incidence in adulthood through an epigenetic pathway (Greathouse et al. 2012).

Data from two large epidemiologic cohorts used to evaluate the relationship between soy formula feeding during infancy and fibroid risk have provided some support for an association in women (D’Aloisio et al. 2010, 2012; Wise et al. 2012). However, given the prevalence of undiagnosed fibroids (Baird et al. 2003), those studies were limited by relying on the self-report of clinical fibroid diagnosis. The purpose of the present analyses was to evaluate infant soy formula feeding in relation to fibroid prevalence, number, and size, using data from a cohort of young, African-American women who were screened by ultrasound for fibroids at study enrollment.

## Methods

### Study Population

We conducted the present analyses using enrollment data from the National Institute of Environmental Health Sciences (NIEHS) Study of Environment, Lifestyle & Fibroids (SELF). The design of SELF, the recruitment strategy, and enrollment data collection have been previously described (Harmon et al. 2013; Moore et al. 2014). Briefly, SELF is an ongoing 5-year prospective cohort study designed to identify incident fibroids and risk factors for fibroid onset and growth. Because African-American women experience greater morbidity from fibroids and have an earlier onset of disease development than do white women (Baird et al. 2003; Kjerulff et al. 1996; Laughlin et al. 2009), SELF is being conducted among 1,696 African-American women ages 23 to 34 years. Study participants were recruited from the Detroit, Michigan, area in collaboration with the Henry Ford Health System. The goal of the recruitment strategy was to saturate the area with information about the study through media advertisements, flyers, letters to users of the healthcare system, a website, and a presence at community events. Women eligible for the study were those with an intact uterus, no prior clinical diagnosis of uterine fibroids, and a willingness to provide information that could be used for tracing and cohort follow-up. Exclusion criteria included a prior diagnosis of any of the following conditions that required treatment with medication: Grave’s disease, Sjogren’s syndrome, scleroderma, multiple sclerosis, or lupus, as well as a prior diagnosis of cancer that was treated with radiation or chemotherapy. SELF study participants were enrolled between January 2010 and December 2012 and participated in several study activities at enrollment. These activities included attending a clinic visit in which ultrasound was performed and anthropometric measurements were taken, completing a computer-assisted telephone interview and web-based questionnaire, as well as several self-administered questionnaires. The institutional review boards at NIEHS and Henry Ford Health System approved the conduct of the SELF study and each participant provided informed consent before enrollment.

### Exposure Ascertainment

The exposure of interest in the present analysis—soy formula feeding during infancy—was ascertained from the questionnaire on early life that was given to participants at the clinic visit and returned by mail. The questionnaire was designed so that the participant could interview her mother to obtain information on early-life characteristics and exposures. Participants who reported not being able to interview their mothers were given another version of the form that elicited the same information, and each was encouraged to seek assistance from other relatives or her mother’s friends for its completion. The early-life questionnaire was completed and returned by 1,598 SELF participants (94%). The questions on infant soy formula feeding in the questionnaire included whether the participant was ever fed soy formula as an infant (yes, no), the duration of soy formula feeding (< 1 month, 1–3 months, 4–6 months, > 6 months), and whether the soy formula feeding was initiated within the first 2 months after birth (yes, no). Because of small numbers when additionally categorizing the exposure by duration and timing, we present data on 1,553 women who provided information on the dichotomous exposure to infant soy formula feeding (198 ever exposed, 1,355 never exposed). Eighty-nine percent of these participants received the assistance of their mothers when completing the questionnaire.

### Outcome Ascertainment

The entire cohort of SELF participants (*n* = 1,696) were screened for uterine fibroids by transvaginal ultrasound and additionally with a transvesical approach if necessary. Ultrasound examinations were conducted by sonographers at three sites within the Henry Ford Health System. Study sonographers had at least 3 years of experience in gynecologic ultrasound and received formal training for the study. Still and video images were archived for each ultrasound examination and 29.5% were reviewed by the head sonographer for quality control. Using the head sonographer’s examination as the gold standard, the initial determination of fibroid status had a sensitivity of 98.3% and specificity of 99.6%. The presence of visualized fibroids or questionable fibroids with a dimension of ≥ 0.50 cm in any of three planes was documented and up to 10 fibroids were counted. Additionally, the 6 largest fibroids were located and measured three separate times during the examination and each time the longitudinal, anterior–posterior, and transverse planes were measured. The diameter of the largest fibroid was estimated using the average maximum dimension from the three measurements for the fibroid. To estimate the total volume of all fibroids, we calculated the volume of each fibroid using the formula for the volume of a prolate ellipse (π/6 × longitudinal diameter × anterior–posterior diameter × transverse diameter). We averaged the volumes for each fibroid across multiple measurements, and summed the average volumes. Among our study sample of 1,553 participants, 5 women had a single, questionable fibroid that the examining sonographer was unable to measure in all three dimensions. These women were categorized as having a fibroid; however, fibroid characteristics were not analyzed.

For the present analyses, the outcomes considered were the presence or absence of uterine fibroids and, among women with fibroids, the number of fibroids detected (1 fibroids, ≥ 2 fibroids), diameter of the largest fibroid (continuous, centimeters), and the total volume of all fibroids (continuous, cubic centimeters).

For use in secondary analyses, we categorized the diameter of the largest fibroid (< 2 cm, ≥ 2 cm), and the total volume of all fibroids (< 5.0 cm^3^, ≥ 5.0 cm^3^) using as the cut point the upper tertile (66th percentile) of the distribution of these characteristics in our data. We selected the cut point in this manner given that the detected fibroids in our study sample were generally smaller than those reported in prior fibroid screening studies (Baird et al. 2003; Myers et al. 2012) due to the relatively young age of our participants and the lack of prior fibroid diagnosis.

### Ascertainment of Participant and Maternal Characteristics

Characteristics of the participant at enrollment, the participant as an infant, and her mother when she was pregnant with the participant were primarily ascertained by questionnaire and interview. Participant height and weight were measured at the clinic visit. Other participant characteristics included age at ultrasound, parity, age at menarche, educational status, total annual household income, smoking and alcohol consumption history, and body mass index (BMI). The characteristics of the participant as an infant included birth weight, gestational age at birth, multiple gestation, first-born status, and whether the participant was ever breastfed and the number of months the participant was breastfed. The highest educational level of mother or primary caregiver when the participant was age 10 years and economic status during the participant’s childhood were also ascertained. Childhood economic status was assessed by asking participants to characterize their household income while growing up (well off, middle income, low income, poor). Data collected on the characteristics of the participant’s mother included age at delivery, smoking during pregnancy, gestational or preexisting diabetes, and pregnancy-related hypertension including preeclampsia.

### Statistical Analyses

We descriptively compared participants who were ever fed soy formula with those never fed soy formula as infants, considering demographic and lifestyle factors, infant characteristics, and maternal factors. We used log-binomial regression (Barros and Hirakata 2003; Blizzard and Hosmer 2006) to estimate the adjusted prevalence ratio (aPR) and 95% confidence interval (CI) for the association between ever feeding of infant soy formula and the presence of any uterine fibroids among our entire study sample (*n* = 1,553) as well as the number of fibroids among women with distinct fibroids (*n* = 340). Among women with distinct fibroids, we also estimated the percent difference in the diameter of the largest fibroid and the total fibroid volume between exposed and unexposed participants using multivariable linear regression. The percent difference was determined by modeling the natural log of the continuous fibroid size variables and exponentiating the regression beta coefficients and 95% CIs. Given that the exposure–disease relationship may not be linear or monotonic, we evaluated categories of the largest fibroid diameter (< 2 cm, ≥ 2 cm) and total fibroid volume (< 5, ≥ 5 cm^3^) in relation to soy formula feeding among women with fibroids using log-binomial regression in a secondary analysis.

We identified variables necessary for adjustment *a priori*, using a conceptual framework for the exposure–disease relationship informed by previous studies on risk factors for fibroids and predictors of infant soy formula feeding (Adgent et al. 2012; D’Aloisio et al. 2010, 2012; Wise et al. 2012). Given that age is a strong predictor of fibroid prevalence, all analyses were adjusted for age of participant at ultrasound (continuous). In addition to age-adjusted estimates, we calculated multivariable-adjusted estimates, further adjusting for participant birth weight (< 2,500 g, ≥ 2,500 g) and maternal characteristics of smoking (yes, no), education [≤ high school diploma or general equivalency diploma (GED), some college, associate’s degree, or higher degree], and any report of gestational diabetes, preexisting diabetes, gestational hypertension or preeclampsia while pregnant with the participant (yes, no). We did not consider participants’ adult factors for adjustment given that these factors could be affected by exposure and therefore would not meet the criteria for confounding.

We conducted several sensitivity analyses. First, we repeated the analyses adjusting for breastfeeding (ever, never). Second, we adjusted for family history of fibroids based on report of fibroid diagnosis for the participant’s mother or sisters (including maternal half-sisters) (yes/no). Third, we conducted an analysis requiring at least 1 month of soy formula feeding to be considered exposed (≥ 1 month vs. never). Fourth, we repeated the analyses restricting the study population to women who were singleton infants, born within 2 weeks of the estimated delivery date and who weighed ≥ 2,500 g at birth. Last, we performed a sensitivity analysis to evaluate the potential influence of selection bias due to the exclusion of women with a prior clinical diagnosis of fibroids as described in more detail in “Results.”

The statistical analyses were performed using STATA version 12.0 (StataCorp, College Station, TX), and statistical significance was determined using the significance level of α = 0.05. In our secondary multivariable-adjusted analysis evaluating categories of the largest fibroid diameter and total fibroid volume using log-biniomial regression, we used the “search” option within the glm command in Stata to address model nonconvergence and to search for appropriate starting values for model parameters (Barros and Hirakata 2003). The estimates from all of our log-binomial regression analyses were similar to those produced using Poisson regression with robust variance (data not shown). In addition, the estimates we obtained using multivariable linear regression did not substantially differ from those using robust regression with iteratively re-weighted least squares (data not shown).

## Results

The adult characteristics of participants with data on infant soy formula feeding were generally similar to those of the entire SELF cohort (data not shown). Among the 1,553 SELF participants in the present analyses, 13% (*n* = 198) of participants reported ever being fed infant soy formula. A high proportion of the participants were fed soy formula for > 6 months (53%, *n* = 188 with available data) and the feeding was initiated within 2 months after birth for 58% (*n* = 186 with available data). The participants were born during the years 1975–1989, when all soy formulas in the United States contained the same soy component, isolated soy protein (Fomon 2001). Fewer than 1% of the participants were born outside the United States. We observed that participants who were ever fed soy formula as infants were generally similar demographically to those never fed soy formula, although a slightly greater percentage of soy-formula fed participants were 23–25 years of age at ultrasound (28% vs. 22%, Table 1).

**Table 1:** Characteristics of participants by soy formula feeding exposure during infancy in the Study of Environment, Lifestyle & Fibroids (SELF), 2010–2012 [*n* (%)].

Characteristic	Ever fed soy formula (*n* = 198)	Never fed soy formula (*n* = 1,355)
Adult characteristics
Participant age at ultrasound (years)
23–25	56 (28)	298 (22)
26–28	50 (25)	333 (25)
29–31	51 (26)	370 (27)
32–35	41 (21)	354 (26)
Parity (number of births)
0	85 (43)	531 (39)
1	47 (24)	350 (26)
2	35 (18)	241 (18)
≥ 3	31 (16)	233 (17)
Age at menarche (years)
≤ 10	38 (19)	248 (18)
11	46 (23)	260 (19)
12	52 (26)	361 (27)
13	33 (17)	230 (17)
≥ 14	29 (15)	256 (19)
Education
≤ High school or GED	36 (18)	299 (22)
Some college or Associate/technical degree	100 (51)	676 (50)
Bachelor’s, Master’s, or doctoral degree	62 (31)	379 (28)
Missing	0	1
Total annual household income (US$)
< 20,000	82 (42)	621 (46)
20,000–50,000	73 (37)	501 (37)
> 50,000	41 (21)	224 (17)
Missing	2	9
Smoking status
Never	152 (77)	994 (73)
Former	16 (8)	99 (7)
Current	30 (15)	262 (19)
Alcohol use
Low/never	52 (26)	359 (26)
Moderate	61 (31)	454 (34)
Heavy	85 (43)	542 (40)
Body mass index (kg/m^2^)
< 18.5	4 (2)	9 (1)
18.5 to < 25.0	28 (14)	263 (19)
25.0 to < 30.0	45 (23)	273 (20)
30.0 to < 35.0	40 (20)	261 (19)
≥ 35.0	81 (41)	549 (41)
Infant characteristics
Birth weight (g)
< 2,500	29 (15)	181 (13)
2,500 to < 3,500	113 (58)	853 (63)
≥ 3,500	55 (28)	311 (23)
Missing	1	10
Born a week or more before due date
No	140 (72)	984 (75)
Yes	54 (28)	325 (25)
Yes, born 1–2 weeks early	25 (13)	186 (14)
Yes, born ≥ 3 weeks early	26 (13)	121 (9)
Yes, unknown number of weeks born early	3 (2)	18 (1)
Missing	4	46
Multiple gestation
No	192 (97)	1,314 (97)
Yes	6 (3)	41 (3)
First born
No	111 (57)	776 (58)
Yes	84 (43)	563 (42)
Missing	3	16
Duration breastfed (months)
Never breastfed	105 (53)	950 (71)
Any breastfeeding	93 (47)	396 (29)
< 1	18 (9)	64 (5)
1–3	34 (17)	105 (8)
4–6	21 (11)	78 (6)
> 6	16 (8)	121 (9)
Duration unknown	4 (2)	28 (2)
Missing	0	9
Childhood characteristics
Highest educational level of mother or primary caregiver when respondent was age 10 years
≤ High school or GED	72 (36)	643 (48)
Some college or Associate/technical degree	93 (47)	557 (41)
Bachelor’s/Master’s/doctoral degree	33 (17)	153 (11)
Missing	0	2
Economic status during participant’s childhood^*a*^
Poor	2 (1)	69 (5)
Low income	72 (36)	480 (35)
Middle income	110 (56)	702 (52)
Well off	14 (7)	103 (8)
Missing	0	1
GED, general equivalency diploma.^***a***^Based on participant’s reporting of one of the listed economic status categories to characterize her household income while growing up.		

With regard to the characteristics of the participant as an infant, soy formula–fed participants compared with non-soy–fed participants tended to weigh more at birth (28% vs. 23% were ≥ 3,500 g), to be born ≥ 3 weeks early (13% vs. 9%), and to be breastfed as infants for at least a short time (37% vs. 19% were breastfed ≤ 6 months) (Table 1). However, few participants were breastfed for > 6 months in either exposure group (8% and 9%). Additionally, soy formula–fed participants were more likely to have mothers or primary caregivers with at least some college education when the participant was age 10 years than participants not fed soy formula (64% vs. 52%).

As for maternal characteristics, a greater proportion of soy formula–fed participants than those not fed soy formula had mothers who were older at delivery (25% vs. 18% were ages ≥ 30 years) and who experienced pregnancy-related hypertension when pregnant with the participant (21% vs. 11%, Table 2).

**Table 2:** Characteristics of mother when pregnant with the participant by participant soy formula exposure during infancy in the Study of Environment, Lifestyle & Fibroids (SELF), 2010–2012 [*n* (%)].

Characteristic	Ever fed soy formula(*n *= 198)	Never fed soy formula(*n *= 1,355)
Age at delivery (years)
12–19	35 (18)	312 (23)
20–24	63 (32)	439 (32)
25–29	52 (26)	362 (27)
30–34	35 (18)	174 (13)
35–52	13 (7)	68 (5)
Maternal smoking
No	147 (74)	994 (73)
Yes	51 (26)	361 (27)
Gestational or preexisting diabetes
No	181 (93)	1,274 (95)
Yes	14 (7)	65 (5)
Missing	3	16
Pregnancy-related hypertension or preeclampsia
No	155 (79)	1,175 (89)
Yes	40 (21)	148 (11)
Missing	3	32

Uterine fibroids were detected in 22% of our sample at enrollment. Among women with fibroids, the majority had one fibroid [median, 1; interquartile range (IQR): 1–2]. The distributions of the largest fibroid diameter and total fibroid volume were right-skewed; the median diameter of the largest fibroid was 1.7 cm (IQR: 1.1–2.8 cm; minimum 0.5 cm, maximum 10.9 cm) and the median of the total fibroid volume was 1.9 cm^3^ (IQR 0.5–11.0 cm^3^; minimum 0.04 cm^3^, maximum 605.0 cm^3^).

The crude prevalence of fibroids in soy formula–fed women was slightly lower than in unexposed women (20% vs. 23%), but exposed women tended to be younger than unexposed women; after either age- or multivariable adjustment there was no association between soy formula feeding and fibroid prevalence (aPR = 0.9; 95% CI: 0.7, 1.3, Table 3). Nor was there an association with tumor number among women with fibroids (Table 4). However, among women with fibroids, those fed soy formula as infants had significantly larger fibroids than unexposed women (Table 5). On average, soy formula feeding was associated with a 32% increase in the diameter of the largest fibroid (95% CI: 6%, 65%) and a 127% increase in total tumor volume (95% CI: 12%, 358%).

**Table 3:** Adjusted prevalence ratio (PR) and 95% CI for the association between infant soy formula feeding and ultrasound-detected fibroids at enrollment, Study of Environment, Lifestyle & Fibroids (SELF), 2010–2012.

Exposure	Fibroids[*n* (%)]	No fibroids[*n* (%)]	Age-adjusted [PR (95% CI)]	Multivariable-adjusted^*a*^ [PR (95% CI)]
Soy formula
Never fed	306 (89)	1,049 (87)	1.0 (reference)	1.0 (reference)
Ever fed	39 (11)	159 (13)	1.0 (0.7, 1.3)	0.9 (0.7, 1.3)
^***a***^Adjusted for participant age and birth weight and maternal smoking, education, and any pregnancy complication of preexisting diabetes, gestational diabetes, pregnancy-related hypertension or preeclampsia.				

**Table 4:** Adjusted prevalence ratio (PR) and 95% CI for the association between infant soy formula feeding and number of fibroids, among women with ultrasound-detected distinct fibroids at enrollment (*n *= 340), Study of Environment, Lifestyle & Fibroids (SELF), 2010–2012.

Exposure	Fibroid number		Age-adjusted [PR (95% CI)]	Multivariable-adjusted^*a*^ [PR (95% CI)]
	≥ 2 Fibroids [*n* (%)]	1 Fibroid [*n* (%)]		
Soy formula
Never fed	112 (88)	190 (89)	1.0 (reference)	1.0 (reference)
Ever fed	15 (12)	23 (11)	1.1 (0.7–1.6)	1.0 (0.7–1.6)
^***a***^Adjusted for participant age and birth weight and maternal smoking, education, and any pregnancy complication of preexisting diabetes, gestational diabetes, pregnancy-related hypertension or preeclampsia.				

**Table 5:** Percent difference in fibroid size and 95% CIs comparing women ever fed and women never fed soy formula, among women with ultrasound-detected fibroids (*n *= 340), Study of Environment, Lifestyle & Fibroids (SELF), 2010–2012.

Exposure	*n* (%)	Diameter of the largest fibroid (cm)			Total volume of fibroids (cm^3^)		
		Median (IQR)	Age-adjusted% difference (95% CI)	Multivariable-adjusted^*a*^ % difference (95% CI)	Median (IQR)	Age-adjusted % difference (95% CI)	Multivariable-adjusted^*a*^ % difference (95% CI)
Soy formula
Never fed	302 (89)	1.6 (1.1, 2.6)			1.7 (0.5, 9.3)
Ever fed	38 (11)	2.3 (1.5, 3.8)	32 (6, 65)	32 (6, 65)	5.4 (1.0, 32.7)	126 (12, 355)	127 (12, 358)
Abbreviations: % difference, percent difference; CI, confidence interval; IQR, interquartile ratio. The percent difference was estimated using multivariable linear regression with the natural log of the fibroid size variable as the dependent variable. The regression beta coefficient and 95% CI were exponentiated and the percent difference was calculated using the formula [(e^β^ – 1) × 100]. ^***a***^Adjusted for participant age and birth weight and maternal smoking, education, and any pregnancy complication of preexisting diabetes, gestational diabetes, pregnancy-related hypertension or preeclampsia.							

In our secondary analyses of fibroid size among those with fibroids, soy formula feeding was significantly associated with a fibroid diameter ≥ 2 cm (aPR = 1.6; 95% CI: 1.1, 2.2, Table 6) and a total fibroid volume ≥ 5 cm^3^ (aPR = 1.6; 95% CI: 1.1, 2.4, Table 7).

**Table 6:** Adjusted prevalence ratio (PR) and 95% CI for the association between infant soy formula feeding and categories of the largest fibroid diameter (< 2, ≥ 2 cm), among women with ultrasound-detected distinct fibroids at enrollment (*n *= 340), Study of Environment, Lifestyle & Fibroids (SELF), 2010–2012.

Exposure	Diameter of the largest fibroid		Age-adjusted PR (95% CI)	Multivariable-adjusted^*a*^ PR (95% CI)
	≥ 2 cm, *n* (%)	< 2 cm, *n* (%)		
Soy formula
Never fed	108 (83)	194 (92)	1.0 (reference)	1.0 (reference)
Ever fed	22 (17)	16 (8)	1.7 (1.3–2.3)	1.6 (1.1–2.2)
^***a***^Adjusted for participant age and birth weight and maternal smoking, education, and any pregnancy complication of pre-existing diabetes, gestational diabetes, pregnancy-related hypertension or pre-eclampsia.				

**Table 7:** Adjusted prevalence ratio (PR) and 95% CI for the association between infant soy formula feeding and categories of the total fibroid volume (< 5, ≥ 5 cm^3^), among women with ultrasound-detected distinct fibroids at enrollment (*n *= 340), Study of Environment, Lifestyle & Fibroids (SELF), 2010–2012.

Exposure	Total fibroid volume		Age-adjusted[PR (95% CI)]	Multivariable-adjusted^*a*^[PR (95% CI)]
	≥ 5 cm^3^ [*n* (%)]	< 5 cm^3^ [*n* (%)]		
Soy formula
Never fed	96 (83)	206 (92)	1.0 (reference)	1.0 (reference)
Ever fed	19 (17)	19 (8)	1.7 (1.2–2.4)	1.6 (1.1–2.4)
^***a***^Adjusted for participant age and birth weight and maternal smoking, education, and any pregnancy complication of preexisting diabetes, gestational diabetes, pregnancy-related hypertension or preqeclampsia.				

Our estimates for the associations between infant soy formula feeding and fibroid outcomes did not appreciably change when we further adjusted for breastfeeding (ever/never) or family history of fibroids (yes/no) (data not shown). Our results were also essentially unchanged when we required at least 1 month of soy formula feeding to be considered exposed (excluding 24 participants fed soy formula < 1 month), or when we restricted the analyses to women who were singleton infants, born within 2 weeks of the estimated delivery date and who weighed ≥ 2,500 g at birth (*n* = 1,208, 148 exposed and 1,060 unexposed; data not shown).

We performed a sensitivity analysis to investigate whether the observed association between soy formula feeding and larger fibroid size could plausibly be attributable to selection bias resulting from our exclusion of women with previously diagnosed fibroids. Given that we observed an association with larger fibroid size, but not fibroid prevalence, soy formula–fed women with small fibroids would need to have been selectively excluded for this bias to occur. This could be possible if soy formula feeding caused symptoms, such as heavy bleeding, that led to the incidental detection of fibroids, because incidentally detected fibroids are more likely to be smaller than symptomatic fibroids (Wegienka et al. 2003). We first calculated the estimated number of “missing” women with fibroids. Then we compared the proportion of small fibroids (< 2 cm in diameter) that would be needed among the “missing” soy formula–fed participants to that expected (Baird et al. 2003), if no association truly exists between soy formula feeding and fibroid size.

For this calculation, we made the following assumptions based on data in the literature: *a*) 30% of black women ages 23–34 years who have ultrasound detectable fibroids have already been diagnosed (Myers et al. 2012); *b*) 20% of the black women with a previous fibroid diagnosis have small fibroids (< 2 cm in diameter) (Baird et al. 2003); and *c*) 13% of women with a previous diagnosis of fibroids were soy formula fed (same frequency as in SELF).

We calculated that we were “missing” 146 women with a prior fibroid diagnosis (30% of 486 = 146; 486 – 146 = 340). Of the 146 “missing” from our data set, 29 would have had small fibroids (20% of 146), and the remaining 117 would have had large fibroids. Additionally, 19 would have been soy formula fed (13% of 146), and the remaining 127 would not have been soy formula fed. Using these marginal numbers in a two-by-two table, we estimated that the majority (63%) of those fed soy formula as infants and “missing” from SELF would have had to have small fibroids to produce a relative risk of 1.0 (no association). This proportion (63%) is more than three times greater than the expected proportion of 20% (Baird et al. 2003) and suggests that it is unlikely that the exclusion of women with previously diagnosed fibroids resulted in selection bias strong enough to produce the association we observed between soy formula feeding and larger fibroids.

## Discussion

In this cohort of young African-American women, soy formula feeding was not associated with fibroid prevalence, but among those with fibroids, women fed soy formula as infants had fibroids that were larger in diameter and larger in total volume than unexposed women.

Given the postnatal development of the myometrium (Valdes-Dapena 1973), infancy may be a susceptible time for exposure to exogenous hormones. Infants can be highly exposed to phytoestrogens in soy formula, particularly if soy formula is the exclusive source of nutrition [reviewed by McCarver et al. (2011)]. Measurement of the phytoestrogen genistein in the urine of soy formula–fed infants [based on 125 samples from 54 infants who contributed between 1 and 4 samples during their first 12 months of life (Umbach DM, personal communication)] showed concentrations more than two orders of magnitude greater than those of children ages 6–11 years in the National Health and Nutrition Examination Survey sample (geometric mean concentrations, 5,891 μg/L and 33.8 μg/L, respectively) (Cao et al. 2009; CDC 2008). Given that the phytoestrogen concentrations are high enough in soy formula–fed infant girls to elicit an estrogenic response in vaginal tissue marked by the increase in the proportion of mature superficial epithelial cells that arise under the influence of estrogen (Adgent et al. 2014), it is plausible that these concentrations may also disrupt myometrial development.

Results from an experimental study suggest a pathway by which postnatal exposure to genistein might lead to greater myometrial sensitivity to estrogen and increased leiomyoma incidence in adulthood (Greathouse et al. 2012). That study used an Eker rat model that is genetically predisposed to the development of uterine leiomyomas and administered 50 mg/kg body weight of genistein by subcutaneous injection which in mice models produces serum genistein concentrations similar to that observed in rats exposed to dietary genistein and in infants fed soy formula (Doerge et al. 2002). This Eker rat study showed that genistein exposure on postnatal days 10–12—the developmental equivalent to when a human is born (Quinn 2005)—could reprogram developing myometrial tissue by activating estrogen receptor signaling pathways in the uterus, leading to epigenetic histone modifications. The investigators further demonstrated that these epigenetic changes persisted through age 16 months when adult rats approach reproductive senescence (Quinn 2005), and resulted in hypersensitive estrogen-responsive myometrial gene expression and increased leiomyoma incidence (Greathouse et al. 2012).

Although we did not observe an association between soy formula feeding and increased fibroid prevalence, we did observe that women fed soy formula as infants had larger fibroids than unexposed women. It is possible that larger fibroid size, rather than increased prevalence, may be the first detectable consequence of exposure in young women. In an Eker rat study of early-life diethylstilbestrol (DES) treatment, those treated compared to untreated had larger fibroids, but not greater fibroid prevalence, when examined in their reproductive prime (Cook et al. 2005). It was not until the animals were approaching reproductive senescence that early-life treatment was associated with increased fibroid prevalence (Cook et al. 2005). Thus, the association between soy formula feeding and increased fibroid prevalence in women may be detectable at an older age when fibroids are more prevalent, providing power to detect the association.

The association between soy formula feeding and fibroids has been previously examined in two large epidemiologic cohorts of women (D’Aloisio et al. 2010, 2012; Wise et al. 2012). Using baseline data from the NIEHS Sister Study, D’Aloisio et al. (2012) reported that self-reported clinically diagnosed fibroids were associated with a history of soy formula feeding for black [risk ratio (RR) = 1.26; 95% CI: 0.83, 1.89, *n* = 96 definitely/probably exposed, *n* = 2,486 definitely not/probably not exposed] and white women (RR = 1.33; 95% CI: 1.08, 1.64, *n* = 857 definitely/probably exposed, *n* = 22,061 definitely not/probably not exposed). The authors restricted self-reported fibroid diagnoses to those that occurred on or before age 30 years for black participants or 35 years for white participants, to minimize outcome misclassification given the increased prevalence of undiagnosed fibroids as women age (Baird et al. 2003). The other large prospective cohort study, the Black Women’s Health study, followed premenopausal participants by postal questionnaire every 2 years for new self-reported clinical diagnoses of fibroids (Wise et al. 2012). Wise et al. (2012) reported no overall association between soy formula feeding and fibroid risk. However, for young women (analysis of 1,254 exposed person-years and 14,874 unexposed person-years contributed when < 30 years old), the authors reported an incident rate ratio of 1.28 (95% CI: 0.91, 1.79) (Wise et al. 2012).

The results from the two prior epidemiologic cohorts and the present analyses may be more consistent than first appears. Given that clinically diagnosed fibroids tend to be larger than undiagnosed fibroids (Baird et al. 2003) and that the prior studies included as noncases women with smaller, undiagnosed fibroids, all of the studies may be detecting the relationship between soy formula feeding and larger fibroid size.

Despite the detailed ultrasound data that our study brought to bear on this research question, there is concern about possible selection bias. By design, SELF excluded women with a prior diagnosis of fibroids. Thus, if the reason for prior fibroid diagnosis was related to soy formula feeding, then our results may have been biased by selection.

An artifactual association between soy formula feeding and fibroid size might arise if soy formula–exposed women with small fibroids were more likely to be clinically diagnosed and thus excluded, resulting in a higher proportion of soy formula–exposed women with large fibroids in the study sample. There are data suggesting that this might be plausible. Researchers who compared reproductive characteristics of young adults that had been fed soy formula with those fed cow’s milk formula as infants found that women fed soy formula reported more menstrual pain than those fed cow’s milk (Strom et al. 2001). If soy formula exposure leads to symptoms that result in a clinical work-up, it may be that small fibroids are more likely to be incidentally diagnosed in women fed soy formula as infants, and such women would be excluded from our study. However, for the preponderance of large fibroids to occur among those exposed to infant soy formula in our study, we estimated in our sensitivity analysis that, among women excluded from SELF due to a prior clinical fibroid diagnosis, > 60% of those exposed to soy formula would have had to have small fibroids (< 2 cm diameter) for the observed association between exposure and fibroid size to be an artifact of selection bias alone. Given that this proportion is more than three times the proportion of small fibroids (20%) observed in a sample of black women ages 35–49 years with previously diagnosed fibroids (Baird et al. 2003), this suggests that this scenario is unlikely.

Alternatively, selection may have resulted in an underestimate of the association. If soy formula feeding does increase the size of fibroids, as our results suggest, and large fibroids tend to be more symptomatic or palpable on examination, leading to clinical work-up and fibroid diagnosis, then exposed women with large fibroids would have been selectively excluded from SELF. In this scenario, the association between soy formula feeding and fibroid size would be stronger than we estimated. The next 5 years of prospective data on fibroid incidence and tumor size measurements that will be collected in SELF should help resolve this issue.

Similar to the prior epidemiologic studies, the present analyses were limited by the retrospective ascertainment of soy formula exposure. However, 89% of SELF participants obtained the soy formula feeding information by directly asking their mothers. Studies conducted around the time the SELF cohort was born (years 1975–1989) suggest that mothers frequently initiated the decision to change formula (Polack et al. 1999) and more often changed to a special formula, such as soy formula, for symptoms such as colic and excessive crying that may be memorable (Forsyth et al. 1985). The expectation that mothers would be good sources of information on infant feeding is supported by the similarity in the prevalence of soy formula feeding in SELF (13%) to the estimate we calculated (~ 11%) using data on formula feeding during the 1980s in the United States reported by Fomon (1987). Hence, the mothers of the cohort of young SELF participants are likely to be good reporters, and the misclassification of exposure in the present analyses may have been minimal.

In the present analyses, we were not able to investigate fibroid prevalence, number, and size in relation to exclusive soy formula feeding; this aspect of soy formula feeding was not collected in SELF. Given that we would expect exclusive soy formula feeding to confer the greatest impact on fibroid development, the lack of data may have decreased the sensitivity our study to detect an association with fibroid prevalence. In addition, our study had a relatively small number of exposed women with fibroids, which limited our study power.

Despite these limitations, our study was strengthened by the collection of exposure information from the mothers of 89% of study participants. The other major strength of our study was the ultrasound screening of all participants at enrollment. The ultrasound data substantially minimized outcome misclassification and allowed us to examine soy formula feeding in relation to measured fibroid size.

## Conclusion

In the present analyses, our observation that women exposed to soy formula have larger fibroids than unexposed women provides further support for persistent effects of early life phytoestrogen exposure on the uterus.
